# Implementation of Riding in the Moment™: the crucial role of implementer preparedness and satisfaction in an adaptive horseback riding program for older adults with dementia

**DOI:** 10.3389/frhs.2026.1799552

**Published:** 2026-07-27

**Authors:** Benazir Meera, Melissa Hiatt, Abby Hays, Sarah Punshon, Jeanette Reffstrup Christensen, Beth Fields

**Affiliations:** 1 Department of Exercise Sciences and Recreation, City University of New York—Lehman College, Bronx, NY, United States; 2 Department of Kinesiology, University of Wisconsin-Madison, Madison, WI, United States; 3 Geriatric Research Education and Clinical Center (GRECC), William S Middleton Memorial Veterans' Hospital, Madison, WI, United States; 4 Research Unit for General Practice, Aarhus, Denmark; 5 DRIVEN – Danish Centre for Motivational and Behavioral Science, University of Southern Denmark, Odense, Denmark; 6 Research Unit for General Practice, Department of Public Health, University of Southern Denmark, Odense, Denmark

**Keywords:** adaptive horseback riding, Alzheimer's disease and dementia, community-based interventions, implementation evaluation, non-pharmacological interventions, staff and volunteer training

## Abstract

**Introduction:**

Riding in the Moment™ (RM) is a community-based, standardized, evidence-informed equine-assisted service program designed to support individuals with Alzheimer's disease and related dementias and their caregivers through social connection, physical activity, and meaningful engagement. Delivered by trained non-clinical personnel, RM's long-term success depends on effective implementation and sustainability in real-world settings. This project explored implementation strategies used to deliver RM, focusing on staff and volunteer preparedness and satisfaction.

**Methods:**

A repeated measures survey design that included closed-ended items and open-ended questions, guided by the RE-AIM framework, was used to explore implementation strategies. Pre-and post-implementation surveys assessed *training satisfaction, perceived preparedness*, and *delivery experience* among RM staff and volunteers. Quantitative data were analyzed using descriptive statistics, while qualitative feedback was organized into key categories. Implementation and fidelity checklists were also completed.

**Results:**

Majority of the participants (95%; *n* = 21) reported overall satisfaction with training and felt prepared to deliver RM. All fidelity checklist items were met, indicating strong adherence to program protocols. Post-implementation, majority of the participants (74%; *n* = 11) were extremely satisfied with RM, and 100% (*n* = 15) stated they would recommend the program. Qualitative feedback emphasized the emotional impact of the experience, value of structured training, and opportunities to improve volunteer engagement and communication.

**Discussion:**

This program evaluation project contributes to understanding early-stage implementation considerations for community-based dementia-specific programs in real-world settings. Continued evaluation is needed to determine how these factors influence long-term implementation, program sustainability, and scalability across broader settings.

## 1 Introduction

Alzheimer's disease and related dementias (ADRD) currently affect over 55 million people globally, with nearly 10 million new cases diagnosed annually and one new diagnosis every three seconds (1). Beyond the core symptoms such as progressive cognitive decline, individuals with ADRD experience behavioral and psychological symptoms such as depression, social withdrawal, and disengagement from daily activities, significantly diminishing their quality of life (QoL) and increasing caregiver burden (2). Given these challenges, there is growing interest in non-pharmacological interventions (NPIs) that foster meaningful engagement, enhance emotional well-being, and promote social interaction within supportive community environments for individuals with ADRD and their caregivers (3, 4).

Community-based NPIs that integrate nature, physical activity, and interpersonal connection have shown promise in improving QoL for individuals with ADRD and their caregivers (5, 6). Among these, equine-assisted services (EAS) have demonstrated benefits in reducing behavioral symptoms and supporting emotional and relational well-being for participants and caregivers (7, 8). Riding in the Moment™ (RM) is one such community-based, standardized, evidence-informed EAS program designed to support individuals with ADRD and their caregivers (9, 10). Grounded in biophilia hypothesis, RM emphasizes social connection, physical activity, and meaningful interactions with animals and people (11). Delivered over eight weeks, participants of RM engage in horseback riding, grooming, and guided interaction with horses in a safe and structured environment (9, 10, 12). RM is delivered by a trained team of non-clinical personnel (staff and volunteers), both of whom play critical roles in facilitating sessions and ensuring participant safety and engagement. Preliminary findings have reported that RM is both feasible and acceptable, with positive effects on QoL of individuals with ADRD and their caregivers (13, 14). However, the long-term success of community-based NPIs like RM depends not only on evidence of efficacy but also on how well the intervention is implemented, adapted, and sustained in real-world settings (15).

Implementation science offers a robust framework for studying these dynamics, moving beyond questions of efficacy to explore *for whom* and *under what conditions* interventions work, and *how* they can be maintained and scaled over time (16). Two early-stage implementation strategies that are critical when interventions are delivered by non-clinical personnel are preparedness and satisfaction (17–19). *Preparedness* refers to an individual's perceived readiness to deliver an intervention, encompassing their knowledge, self-efficacy, and confidence (17), and is influenced by training quality, leadership support, and the perceived relevance of the intervention to local context (17). *Satisfaction* reflects how individuals experience both the training process and program delivery, which can further affect retention, fidelity, and their willingness to continue engagement over time (19). Without intentional focus on implementation strategies, well-designed NPIs may often struggle to move beyond initial pilot phases to achieve lasting impact (20). This challenge is particularly salient for community-based dementia focused NPIs, which rely on non-clinical personnel and are highly sensitive to local organizational and cultural contexts (21).

Therefore, to support the sustained implementation of RM beyond its initial phases and to promote its long-term impact, this program evaluation project explored the implementation strategies used to operationalize RM in practice. Specifically, the purpose of the current project was to explore the preparedness of volunteers and staff to deliver RM, as well as to assess their satisfaction with the training they received and their overall experience implementing RM.

## 2 Methods

### 2.1 Project design and guiding framework

We conducted a repeated measures survey design that included both closed-ended items and open-ended questions. This design is well-suited for examining implementation-related phenomena as they occur in real-world, community-based settings (22). The RE-AIM framework (Reach, Effectiveness, Adoption, Implementation, and Maintenance) offered a structured and pragmatic approach to exploring the implementation processes (23, 24). For the purposes of this project, the authors focused on the *Implementation* domain within RE-AIM, which examines factors influencing the real-world delivery of an intervention including fidelity and adaptations made during implementation. In this project, implementation was specifically evaluated through measures of participant *preparedness* and *satisfaction* with the delivery of RM.

### 2.2 Setting and participants

RM program was originally developed in 2011 and has been iteratively refined over time. However, the present project focuses on implementation at a single site and does not capture participation across the five sites currently delivering the program or over its full history. Accordingly, the project took place at Three Gaits. Therapeutic Horsemanship Center, a nonprofit organization located in Stoughton, WI, that implements EAS for individuals with physical, emotional, and cognitive disabilities. Using purposive and convenience sampling, two participant groups were recruited: volunteers who supported the implementation of RM at Three Gaits Therapeutic Horsemanship Center and staff members responsible for leading the adaptive horseback riding sessions at Three Gaits Therapeutic Horsemanship Center Since the purpose of the project was to support program evaluation, it was determined to be exempt from University of Wisconsin–Madison's Institutional Review Board oversight.

### 2.3 Project procedures

This program evaluation project was conducted from March to May 2024, during which RM was implemented in three distinct stages: Pre-Implementation Stage, Program Implementation Stage, and Post-Implementation Stage.

#### 2.3.1 Pre-implementation stage

To prepare for RM delivery, a collaborative 4-h training workshop was held at Three Gaits Therapeutic Horsemanship Center in March 2024, attended by 19 volunteers, 6 staff members, 3 members of the research team, and one dementia care specialist. This training workshop was co-facilitated by RM research team and established community partners and consisted of three key components:

#### 2.3.1.1 Dementia-friendly training session

Led by a dementia care specialist, this session addressed types and prevalence of ADRD, effective communication strategies, and responses to behavioral symptoms. Experiential activities were incorporated to promote empathy and deepen understanding of the dementia experience.

#### 2.3.1.2 General RM orientation session

Facilitated by research team, this session introduced the overall structure of RM, potential benefits of EAS programs including RM, safety protocols, participant support strategies, roles and responsibilities of staff and volunteers and included hands-on interactive activities.

#### 2.3.1.3 Staff-specific implementation training

This staff-only session focused on the operational aspects of RM delivery, including participant screening and enrollment procedures, fidelity monitoring, safety protocols, and data collection processes.

#### 2.3.2 Program implementation stage

Following the training, an introductory RM session was offered to six caregiver–care recipient dyads at Three Gaits Therapeutic Horsemanship Center. This soft launch served as a small-scale rehearsal of the full 8-week RM program and helped foster early community engagement. A full description of RM is available in the parent papers (11–14).

#### 2.3.3 Post-implementation stage

At the conclusion of the soft launch of RM, feedback was collected from the participants of this project to guide final preparations for the full implementation of RM. This phase allowed for a comprehensive assessment of participants' experiences and provided valuable insights to inform future adaptations and improvements to RM.

### 2.4 Data collection

Data was collected at two key timepoints using Qualtrics-based online surveys: pre-implementation survey following the training workshop, and post-implementation survey after RM concluded (*see* Supplementary Files S2 and S3 *for example survey items*).

#### 2.4.1 Pre-implementation survey

This survey evaluated participants' satisfaction with the training and their preparedness to implement RM. It consisted of quantitative Likert-scale items (e.g., rated from 1 = *Strongly Disagree* to 4 = *Strongly Agree*) and open-ended questions, which allowed for more in-depth, qualitative feedback on their experiences and suggestions for improvement. Additionally, an Implementation Readiness Checklist was completed by RM staff. This checklist included 15 items, each rated on a scale from 1 (*Not at all*) to 5 (*Yes, very well*) to assess components of implementation readiness.

#### 2.4.2 Post-implementation survey

This survey assessed participants' overall satisfaction with the delivery of RM. It included quantitative satisfaction ratings (e.g., ranging from 1 = *Extremely Dissatisfied* to 5 = *Extremely Satisfied*) and open-ended questions to capture participant reflections and suggestions for RM improvement. A Fidelity Checklist was completed by RM staff and reviewed by RM research team. This checklist consisted of 17 items, where staff marked applicable boxes to document adherence to RM delivery standards.

### 2.5 Data analysis

Quantitative data were analyzed using Microsoft Excel. Descriptive statistics (e.g., frequencies, percentages) were used to summarize participant demographics, preparedness, and satisfaction ratings from the close-ended survey items. Responses to open-ended questions were summarized into key categories, with selected illustrative quotes provided to contextualize the findings. Furthermore, the reporting of this project adhered to the STROBE Statement (25) and SQUIRE reporting guidelines (26) was completed (see Supplementary File S1).

## 3 Results

### 3.1 Pre-implementation survey

#### 3.1.1 Participant demographics

Of those who attended, 22 participants completed the pre-implementation phase survey, with 20 providing demographic data. Among them, majority identified as White (*n* = 16; 82%) and female (*n* = 15; 77%), with nearly one-third (*n* = 6; 32%) being retired, and 41% (*n* = 8) held a master's degree or higher. More than half (*n* = 11; 55%) reported being current volunteers at Three Gaits Therapeutic Horsemanship Center.

#### 3.1.2 Satisfaction with training

Majority of the participants (*n* = 21; 95%) reported overall satisfaction with the training workshop. Regarding specific components of the training, an equal number of participants (*n* = 21; 95%) expressed strong agreement/agreement in satisfaction with the length of the workshop, effectiveness of the presenter or facilitator, dementia care content, and content related to RM. Strong agreement/agreement in satisfaction with audiovisual materials was reported by 90% (*n* = 20), and 77% (*n* = 17) were satisfied with training handouts.

#### 3.1.3 Training objectives and content

Ninety percent of participants (*n* = 20) strongly agreed/agreed that the level of content difficulty was appropriate, workshop met their expectations, and the training provided information applicable to implementing RM. Over half (*n* = 12) indicated strong agreement/agreement that the concepts presented were new or novel to them.

#### 3.1.4 Workshop delivery

Most participants (*n* = 21; 95%) strongly agreed/agreed that the activities positively contributed to their learning and that the number of activities was appropriate. Similarly, 95% (*n* = 21) of the participants reported that facilitator interactions significantly impacted their learning experience. Slightly fewer participants (*n* = 20; 90%) strongly agreed/agreed that group discussions following the exercises were beneficial.

#### 3.1.5 Facilitator effectiveness

Ninety-five percent of participants (*n* = 21) strongly agreed/agreed that the facilitator was knowledgeable, maintained their interest throughout the session, and provided satisfactory answers to questions. Additionally, 90% (*n* = 20) noted that the facilitator communicated the material effectively.

#### 3.1.6 Qualitative findings

Overall feedback was positive, with participants describing the training as engaging, informative, and valuable for building understanding of dementia-related sensory and interaction challenges. Participants highlighted hands-on practice, knowledge-based activities, and sensory experience tasks as the most useful components for preparing them to work with the population. Suggestions for improvement included incorporating more scenario-based or applied practice, opportunities to learn directly from family members of individuals with ADRD and video examples of real program sessions to better support applied understanding. *See* Table 1 *for illustrative quotes*.

**Table 1:** Pre implementation survey feedback.

Categories	Illustrative quotes
Perceptions of the training workshop	* “It was great, excited about the program.” ^ “The knowledge check and sensory experience tasks were both engaging and helpful.” ^ “[the sensory experience] allowed a simulated experience to sympathize with the [individuals with] decreased sensory capacities.”
Most helpful materials	* “Hands on practice.” ^ “Hands-on knowledge.” ^ “Practical considerations when interacting with this population.”
Suggestions for improvement	* “It would've been amazing to have a family member of a person with dementia do a little Q&A understanding; everyone is different, but it would allow us to better support, family and participants.” ^ “[include] Video of participant in an actual class.” * “Maybe more scenarios or practice hands on training.”

* Indicates staff responses.

^ Indicates volunteer responses.

#### 3.1.7 Implementation readiness

RM staff including the center director, program manager and lead riding instructor demonstrated a strong sense of preparedness, with 10 out of 15 components on the Implementation Readiness Checklist rated as 5—Yes, Very Well (*See* Table 2).

**Table 2:** Implementation readiness checklist*.

Manual section	Readiness components	1 Not at all	2 A bit	3 We're about 50%	4 Yes, to some extent	5 Yes, very well
Center standards and guidelines	PATH membership and/or accreditation					X
	Necessary equipment and spaces				X	
	Equine selection welfare guidelines					X
	Insurance and legal considerations					X
	Cost estimates of program					X
	Outline session dates and fees					X
Staff and volunteers	Appropriate staff: program director, instructor, equine manager					X
	Recruitment of volunteers					X
	Staff/volunteer training (general and dementia-friendly)					X
	Review safety considerations					X
Marketing and recruitment	Marketing and recruitment of participants					X
Policies and procedures	Review policies and procedures: attendance, attire, safety, cancellation, and discharge				X	
Enrollment and eligibility	Prepare enrollment packets				X	
	Prepare for and organize initial assessment				X	
Curriculum manual	Prepare for and organize first visit				X	

* We recommend that centers use this checklist to determine their readiness for implementing the *Riding in the Moment*^TM^ program.

### 3.2 Post-implementation survey

#### 3.2.1 Participant demographics

A total of 15 participants completed the post-program implementation survey, among whom a majority (*n* = 10; 67%) were volunteers and the rest (*n* = 5; 34%) were staff. Majority identified as White (*n* = 13; 87%) and female (*n* = 14; 93%). Over half (*n* = 8; 54%) reported holding a bachelor's degree or higher, and most of them (*n* = 10; 67%) indicated they were current volunteers at Three Gaits Therapeutic Horsemanship Center.

#### 3.2.2 Satisfaction with program

Participants provided feedback on their satisfaction with RM and the support they received. Among volunteers, 80% (*n* = 8) reported being extremely satisfied, while 20% (*n* = 2) were somewhat satisfied. Among staff, 60% (*n* = 3) were extremely satisfied, 20% (*n* = 1) were somewhat satisfied, and 20% (*n* = 1) reported being extremely dissatisfied.

#### 3.2.3 Continuation of program

Participants expressed strong support for the continuation and future outreach of RM. Among volunteers, 90% (*n* = 9) rated the continuation of RM for individuals with ADRD as extremely important, as did all staff members (*n* = 5). Additionally, all volunteers (*n* = 10) and staff (*n* = 5) indicated they would be extremely likely to recommend the program to friends and family.

#### 3.2.4 Use of program manuals

All staff members (*n* = 5) reported that the program manuals enhanced their understanding of RM content and structure. One staff member indicated their understanding was significantly increased, while the remaining four staff members reported their understanding was somewhat increased.

#### 3.2.5 Qualitative feedback

Overall feedback was positive, with participants describing meaningful emotional engagement, joy, and perceived benefits for individuals with ADRD and their support networks. They emphasized the value of preparatory materials, particularly participant information cards and dementia-related training, in supporting effective communication and interaction during sessions, while also offering suggestions to further enhance engagement and volunteer support, including structured icebreakers, strengthened dementia-friendly communication training, and tangible take-home materials such as photos or memory books. *See* Table 3 *for illustrative quotes*.

**Table 3:** Post implementation survey feedback.

Categories	Illustrative quotes
Perceptions of RM	* “Very positive. Felt it enriched the lives of the participants and the caregivers and the volunteers assisting.” ^ “It [the RM program sessions] truly was my favorite part of the week. Getting to see the joy that it brought participants was unmatched.” ^ “It was incredibly moving to see participants smile for the first time in months or to have them express a memory that was triggered by their experiences.” * “The moments we saw with participants have changed my outlook on life, and it is an experience I will never forget.” * “Just standing next to [a horse] with your hands on them can be a very powerful connection. But watching some of the participants from the first session to the last brought it to a whole new level.” * “Caregivers told us how much their loved ones liked coming here and noticed a positive impact on their lives.” * “It was moving to see the positive impact not only on the participant, but also for their caregivers.”
Most helpful materials	^ “Participant information cards were extremely helpful in breaking the ice and diving deeper into those personal relationships.” ^ “I think the dementia training is the most important so that all volunteers know how to properly interact with the participants.” ^ “Discussion/training beforehand about what types of activities were going to be done helped us assist participants and get them talking about their past experiences.”
Suggestions for improvement	^ “A quick 5-minute icebreaker could smooth out social interactions for the rest of the event.” * “Dedicating some more time to helping volunteers present information in a dementia-friendly way would be another good investment.” ^ “Being able to send participants off with photos or a short-handmade book would be fabulous!”

* Indicates staff responses.

^ Indicates volunteer responses.

#### 3.2.6 Fidelity checklist

RM staff including the center director, program manager and lead riding instructor collaboratively marked off all items, thus indicating complete adherence to the program's delivery standards (see Table 4). Hence, no modifications were made during the program implementation phase.

**Table 4:** Fidelity checklist*.

Category	Checklist item
Center	⊠ *Riding in the Moment*^TM^-trained center is either a PATH, Intl. member or accredited center (see https://pathintl.org/accreditation/). □ *Riding in the Moment*^TM^-trained center follows up on referral with care partners within 1 week of contact (phone or email). ⊠ *Riding in the Moment*^TM^-trained persons and centers are expected to report basic data (See Appendix F-Data Tracking Template in the Implementation Manual) to the Geriatric Health Services Research Lab- UW Madison.
Staff and volunteers	⊠ All *Riding in the Moment*^TM^ therapeutic horseback riding instructors, staff, and volunteers complete recommended dementia training (see Staff and Volunteers section of implementation manual). ⊠ The two therapeutic horseback riding instructors complete a pre-session assessment with persons living with dementia referred to the *Riding in the Moment*^TM^ Program (see Participant Enrollment and Eligibility Screening and Measures sections of implementation manual). ⊠ The two therapeutic horseback riding instructors complete a post-session assessment with participants of the *Riding in the Moment*^TM^ Program (see Participant Enrollment and Eligibility Screening and Measures sections of implementation manual).
Participant and care partners	⊠ Participation by a person living with dementia and their care partner is voluntary. ⊠ All participants and care partners complete enrollment forms for *Riding in the Moment*^TM^ Program prior to the session starting (see Appendix C—Enrollment Packet of implementation manual).
Program	⊠ The *Riding in the Moment*^TM^ Program does not substitute for other long-term services and support programs. ⊠ Minimum number of 4 *Riding in the Moment*^TM^ Program visits per session. □ Maximum of 12 participants per *Riding in the Moment*^TM^ Program visit with only 2 riders mounted at one time. ⊠ Each visit must include a greeting, activities with the horse, thank-you and goodbye, and debrief/reflection (see Chapters 1, 3 and 4 of curriculum manual). ⊠ For visits with 12 participants, there must be a total of 2 therapeutic horseback riding instructors, 1 center staff member, and 21 volunteers - For visits with 6 participants, there must be a total of 2 therapeutic horseback riding instructors, 1 center staff member, 9 volunteers - For visits with less than 3 participants, there must be a total of 1 therapeutic horseback riding instructor, 1 center staff member, and 4 volunteers ⊠ The focus of this adaptive riding program is on promoting quality of life through tailored physical, social, and environmental-based activities (see Chapter 2 of curriculum manual).
Research	⊠ Hearts and Horses and the University of Wisconsin–Madison retains oversight authority to affirm that the *Riding in the Moment*^TM^ Program and the Fidelity Guidelines are being followed (this will most likely be an annual telephone-based check-in). ⊠ All materials are proprietary, and rights are held by Hearts & Horses Inc., and the University of Wisconsin–Madison. ⊠ Centers and other users of the *Riding in the Moment*^TM^ Program are not permitted to use the *Riding in the Moment* trademark and associated logo without prior written permission from Hearts & Horses Inc.

* Fidelity “measures the degree to which an intervention or program is implemented as it was originally prescribed or developed” (44). We have developed these Fidelity Guidelines for *Riding in the Moment™* to be as clear as possible about expectations when implementing and delivering this adaptive riding program. This form can also be found in the implementation manual (before Appendix A) for ease of printing all materials together. To maintain fidelity, follow these guidelines.

## 4 Discussion

This program evaluation project explored the implementation strategies of RM, a community-based, EAS program for individuals with ADRD. Guided by the RE-AIM framework (23, 24), this project reports perceived *preparedness* and *satisfaction* of staff and volunteers with RM's implementation processes.

High levels of satisfaction with both the training and program delivery, as well as fidelity to the intervention, indicate that the implementation strategies employed were successful. Participants reported that the training aligned closely with their perceived responsibilities. The inclusion of interactive elements, particularly dementia simulation, was noted as one of the most impactful components. Such immersive training approaches are consistent with prior research demonstrating that experiential learning can increase awareness, elicit cognitive and emotional responses, and shift perspectives in dementia care by enhancing empathy and understanding, which are foundational to person-centered care (27–29).

The training's multimodal delivery, including didactic instruction, audiovisual materials, and hands-on components, was positively received. Nearly all participants reported satisfaction with the workshop structure, facilitator effectiveness, and relevance of training materials. This is in line with adult learning theory, which emphasizes the importance of interactive, learner-centered training in promoting knowledge retention and application (30). Informal guidance from experienced staff and volunteers also emerged as vital, hence supporting existing literature on the value of peer mentorship in community-based implementation of NPIs (31).

Over half of the participants reported that the training content was new or unfamiliar, thus highlighting the potential of RM to introduce novel and meaningful concepts, even among individuals with prior experience in volunteerism or dementia-related contexts. This finding is notable given that EAS centers often lack dedicated programming or training specifically tailored to individuals with ADRD (32, 43). These findings further underscore the importance of integrating foundational dementia knowledge with applied context-specific content to effectively prepare non-clinical personnel for meaningful implementation roles within community-based dementia focused NPIs (33, 34).

The demographic profile of participants mirrors trends in caregiving and volunteerism in aging services (35, 36). More than half of the participants were already volunteering at Three Gaits Therapeutic Horsemanship Center, which likely contributed to higher program engagement and consistency in their participation in RM. Familiarity with setting and sustained involvement across pre- and post-implementation phases may support long-term feasibility and scale-up of RM (18).

Qualitative feedback further reinforced RM's acceptability (13). Volunteers described RM as emotionally meaningful and rewarding, citing participant joy, memory recall, and connection as central outcomes. Staff echoed these sentiments, emphasizing the transformative nature of human-equine interaction and broader emotional impact of RM. These observations align with prior research supporting the therapeutic potential of EAS for individuals with ADRD (14, 32, 37) as well as the program's indirect benefits for caregivers, who were frequently mentioned as visibly impacted by their loved ones' participation (13, 38).

Both sets of participants offered suggestions to strengthen future iterations of RM, including expanding hands-on components, improving volunteer onboarding, and integrating structured opportunities to share participant progress. Staff recommended clearer activity guidelines and additional training on dementia-friendly communication strategies. These insights support the need for iterative adaptation, continuous feedback loops, and implementation strategies that are responsive to both organizational and individual-level needs for RM (30, 39, 40, 42).

This study is not without limitations, many of which align with considerations outlined in the RE-AIM framework (23). First, it was conducted at a single site with a small sample size, hence limiting reach and generalizability to broader populations or settings. To address this limitation, although RM has been implemented in multiple locations, contextual details such as recommended staffing ratios outlined in the implementation manual of RM may provide insight into delivery expectations and help situate the present findings. Second, data were self-reported and may be influenced by social desirability bias. Third, this study focused on early-stage implementation and did not evaluate maintenance, which is a critical component for understanding sustained impact and scalability. Lastly, although a fidelity checklist was included, its application and assessment were not systematically operationalized or reported, restricting a comprehensive evaluation of program delivery within the RE-AIM framework. Importantly, this evaluation reflects a “soft launch” phase of RM and was guided by the RE-AIM framework to assess implementation outcomes. However, the authors acknowledge that the EPIS (Exploration, Preparation, Implementation, Sustainment) framework may be more appropriate for capturing the dynamic, phased nature of the implementation process (41). Accordingly, findings from this program evaluation will inform the transition to full-scale implementation, during which the EPIS framework will be applied to more systematically guide evaluation across stages. Future work should incorporate more rigorous and transparent fidelity assessment procedures and address the limitations to more comprehensively evaluate RM across implementation phases and outcomes.

## 5 Conclusion

Guided by the RE-AIM framework and focusing on early-stage implementation strategies, this program evaluation project contributes to understanding how community-based dementia-specific NPIs may be implemented in real-world settings. Findings indicate that implementers perceived the training as satisfactory and reported feeling prepared to deliver RM. These results highlight the potential importance of implementers' training needs, communication strategies, and opportunities for feedback in supporting early implementation efforts. Continued evaluation is needed to determine how these factors influence long-term implementation, program sustainability, and scalability across broader settings.

## Data Availability

The original contributions presented in the study are included in the article/Supplementary Material, further inquiries can be directed to the corresponding author.
