# Supplementary material for: Implementation of Riding in the Moment™: the crucial role of implementer preparedness and satisfaction in an adaptive horseback riding program for older adults with dementia
Source: Front Health Serv. 2026 Jul 27;6:1799552. doi: 10.3389/frhs.2026.1799552 (PMC13453946; doi:10.3389/frhs.2026.1799552)
Supplement: Supplementary file 2 [file Table2.docx]

**Riding in the Moment™ Program Training**

**End of Training Evaluation Survey**

*For each of the following statements, circle the number that corresponds to your level of agreement.* **1 =Strongly disagree; 2 = Disagree; 3 = Agree; 4 = Strongly agree.**

**Training Objectives and Content**

| 1. | The content of this workshop met my expectations. | 1 2 3 4 |
| --- | --- | --- |
| 2. | The concepts presented in the workshop were new to me. | 1 2 3 4 |
| 3. | The level of content difficulty was appropriate. | 1 2 3 4 |
| 4. | The workshop provided me with information I can use in the Riding in the Moment™ program. | 1 2 3 4 |

**Workshop Objective**

I am satisfied with the following elements of the workshop:

| 1. | Length of the workshop | 1 2 3 4 |
| --- | --- | --- |
| 2. | Training handouts | 1 2 3 4 |
| 3. | Audiovisual material | 1 2 3 4 |
| *For each of the following statements, circle the number that corresponds to your level of agreement.* **1 =Strongly disagree; 2 = Disagree; 3 = Agree; 4 = Strongly agree.** | | |
| 4. | Presenter/facilitator | 1 2 3 4 |
| 5. | Content covered on dementia care practices | 1 2 3 4 |
| 6. | Content covered on the Riding in the Moment™ program | 1 2 3 4 |

**Workshop Delivery**

| 1. | The activities carried out contributed to my learning. | 1 2 3 4 |
| --- | --- | --- |
| 2. | The number of activities completed was appropriate. | 1 2 3 4 |
| 3. | Exchanges with the facilitator contributed to my learning. | 1 2 3 4 |
| 4. | The group discussions following the exercises contributed to my learning. | 1 2 3 4 |

**Facilitator/Presenter – Dementia Care Practices**

| 1. | The facilitator was knowledgeable regarding the workshop content. | 1 2 3 4 |
| --- | --- | --- |
| 2. | The facilitator communicated clearly. | 1 2 3 4 |
| *For each of the following statements, circle the number that corresponds to your level of agreement.* **1 =Strongly disagree; 2 = Disagree; 3 = Agree; 4 = Strongly agree.** | | |
| 3. | The facilitator gave satisfactory answers to questions. | 1 2 3 4 |
| 4. | The facilitator helped maintain my interest in the workshop. | 1 2 3 4 |

**Facilitator/Presenter – Riding in the Moment™ Program**

| 1. | The facilitator was knowledgeable regarding the workshop content. | 1 2 3 4 |
| --- | --- | --- |
| 2. | The facilitator communicated clearly. | 1 2 3 4 |
| 3. | The facilitator gave satisfactory answers to questions. | 1 2 3 4 |
| 4. | The facilitator helped maintain my interest in the workshop. | 1 2 3 4 |

**Overall Experience**

| 1. | Overall, I was satisfied with this workshop. | 1 2 3 4 |
| --- | --- | --- |

2. What did you like most about this workshop?

3. Do you have any suggestions (content, material, other) to improve this workshop?

4. How has the sensory experience either given you perspective or changed your opinion on interacting with individuals with dementia?
