# Supplementary material for: Implementation of Riding in the Moment™: the crucial role of implementer preparedness and satisfaction in an adaptive horseback riding program for older adults with dementia
Source: Front Health Serv. 2026 Jul 27;6:1799552. doi: 10.3389/frhs.2026.1799552 (PMC13453946; doi:10.3389/frhs.2026.1799552)
Supplement: Supplementary file 3 [file Table3.docx]

Implementation Satisfaction Survey- Staff

Q3 Let's start with some demographic questions, so we can get to know a little bit more about your background and experience.

Q4 What is your age?

Q5 What gender do you identify as?

- Male (1)
- Female (2)
- Non-binary / third gender (3)
- Prefer not to say (4)

Q6 Please specify your race/ethnicity (Select all that apply):

- Native American (1)
- Asian (2)
- Black or African American (3)
- Native Hawaiian or Other Pacific Islander (4)
- Caucasian (5)
- Latino or Hispanic (6)
- Other (7) __________________________________________________
- Prefer not to say (8)

Q7 What is your highest level of education?

- Less than high school diploma (1)
- High School Diploma/GED (2)
- Some college, no degree (3)
- Associate degree (4)
- Bachelor's degree (5)
- Post-graduate degree (6)
- Prefer not to say (7)

Q8 How long have you worked at blinded for review? (please specify years or months)

________________________________________________________________

Q9 What is your primary responsibility at Three Gaits Therapeutic Horsemanship Center Inc.?

- Leadership (1)
- Riding instructor (2)
- Horse and barn care (3)
- Therapist (4)

Q10 Now, we'd like to learn more about your experience with the program. Please answer the following questions.

Q11 Did you attend the dementia training in March at Three Gaits Therapeutic Horsemanship Center Inc.?

- Yes (1)
- No (2)

Q12 How familiar with dementia were you prior to starting the Riding in the Moment^TM^ program?

- Not familiar at all (1)
- Slightly familiar (2)
- Moderately familiar (3)
- Very familiar (4)
- Extremely familiar (5)

Q23 How familiar with dementia were you prior to starting the Riding in the Moment^TM^ program?

- Not familiar at all (1)
- Slightly familiar (2)
- Moderately familiar (3)
- Very familiar (4)
- Extremely familiar (5)

Q13 How satisfied were you with the implementation of the Riding in the Moment^TM^ program at Three Gaits Therapeutic Horsemanship Center Inc?

- Extremely dissatisfied (1)
- Somewhat dissatisfied (2)
- Neither satisfied nor dissatisfied (3)
- Somewhat satisfied (4)
- Extremely satisfied (5)

Q14 Knowing how to implement the Riding in the Moment^TM^ program was straightforward.

- Strongly disagree (1)
- Somewhat disagree (2)
- Neither agree nor disagree (3)
- Somewhat agree (4)
- Strongly agree (5)

Q15 Based on your experience with the Riding in the Moment^TM^ program how important is it to continue this program for the dementia population?

- Not at all important (1)
- Slightly important (2)
- Moderately important (3)
- Very important (4)
- Extremely important (5)

Q16 To what extent did the manuals for the Riding in the Moment^TM^ program increase your understanding of implementation?

- Significantly increased my understanding (1)
- Somewhat increased my understanding (2)
- Did not increase my understanding (3)
- Did not use the manuals (4)
- Did not know about the manuals (5)

Q17 How satisfied are you with data collection support from the Geriatric Health Services Research Lab-UW Madison at the Three Gaits Therapeutic Horsemanship Center Inc?

- Extremely dissatisfied (1)
- Somewhat dissatisfied (2)
- Neither satisfied nor dissatisfied (3)
- Somewhat satisfied (4)
- Extremely satisfied (5)
- Not sure/not applicable (6)

Q18 Considering your experience with Riding in the Moment^TM^, how likely are you to recommend this program to friends/family or colleagues?

- Extremely unlikely (1)
- Somewhat unlikely (2)
- Neither likely nor unlikely (3)
- Somewhat likely (4)
- Extremely likely (5)

Q20 Please describe the safety issues or concerns you had about the program.

________________________________________________________________

________________________________________________________________

Q21 Overall, how would you describe your experience with Riding in the Moment^TM^?

________________________________________________________________

________________________________________________________________

Q22 Do you have any other comments, suggestions, or concerns that can help us to improve Riding in the MomentTM?

________________________________________________________________

________________________________________________________________

________________________________________________________________

**Implementation Satisfaction Survey - Volunteer**

Q3 Let's start with some demographic questions, so we can get to know a little bit more about your background and experience.

Q4 What is your age?

________________________________________________________________

Q5 What gender do you identify as?

- Male (1)
- Female (2)
- Non-binary / third gender (3)
- Prefer not to say (4)

Q6 Please specify your race/ethnicity (Select all that apply):

- Native American (1)
- Asian (2)
- Black or African American (3)
- Native Hawaiian or Other Pacific Islander (4)
- Caucasian (5)
- Latino or Hispanic (6)
- Other (7) __________________________________________________
- Prefer not to say (8)

Q7 What is your highest level of education?

- Less than high school diploma (1)
- High School Diploma/GED (2)
- Some college, no degree (3)
- Associate degree (4)
- Bachelor's degree (5)
- Post-graduate degree (6)
- Prefer not to say (7)

Q8 How long have you worked at blinded for review? (please specify years or months)

________________________________________________________________

Q9 Now, we'd like to learn more about your experience with the program. Please answer the following questions.

Q10 Did you attend the dementia training in March at the blinded for review?

- Yes (1)
- No (2)

Q11 How familiar with dementia were you prior to starting the Riding in the MomentTM program?

- Not familiar at all (1)
- Slightly familiar (2)
- Moderately familiar (3)
- Very familiar (4)
- Extremely familiar (5)

Q12 How satisfied were you with how Riding in the MomentTM was implemented at blinded for review?

- Extremely dissatisfied (1)
- Somewhat dissatisfied (2)
- Neither satisfied nor dissatisfied (3)
- Somewhat satisfied (4)
- Extremely satisfied (5)

Q13 Knowing what to do as a volunteer for the program was straightforward.

- Strongly disagree (1)
- Somewhat disagree (2)
- Neither agree nor disagree (3)
- Somewhat agree (4)
- Strongly agree (5)

Q14 Based on your experience with the Riding in the MomentTM, how important is it to continue this program for the dementia population?

- Not at all important (1)
- Slightly important (2)
- Moderately important (3)
- Very important (4)
- Extremely important (5)

Q15 Considering your experience with Riding in the MomentTM, how likely are you to recommend this program to friends/family or colleagues?

- Extremely unlikely (1)
- Somewhat unlikely (2)
- Neither likely nor unlikely (3)
- Somewhat likely (4)
- Extremely likely (5)

Q18 Overall, how would you describe your experience with Riding in the MomentTM?

________________________________________________________________

Q19 What materials (e.g., dementia training, manuals, participant information cards) did you feel were most beneficial to helping you serve as a volunteer for Riding in the MomentTM?

________________________________________________________________

________________________________________________________________

__

Q20 Do you have any other comments, suggestions, or concerns that can help us to improve Riding in the MomentTM?

________________________________________________________________

________________________________________________________________
